# Non-steroidal anti-inflammatory drugs and their benefits and harms: the challenge of interpreting meta-analyses and observational data sets when balanced data are not analyzed and reported

**DOI:** 10.1186/s13075-015-0650-1

**Published:** 2015-05-21

**Authors:** Lee S. Simon

**Affiliations:** SDG LLC, One Mifflin Place, Suite 400, Cambridge, MA 02138 USA

## Abstract

A multitude of reports have delineated the risks of using non-steroidal anti-inflammatory drugs but have not been totally congruent. Meta-analyses of randomized controlled trials sometimes concur regarding gastrointestinal risk and cardiovascular risk but rarely report a balance of these risks for any one drug. Benefits measured in these studies are usually not reported. Observational data sets, supposedly reflective of ‘real world’ patients, do not always agree with the randomized controlled trial reports. Clinicians need assessments measuring the balance of harms and benefits so that better decisions based on their patients’ unique risk factors can be reached.

Van Walsem and colleagues, in a recent article in *Arthritis Research & Therapy*, performed a network meta-analysis uniquely comparing diclofenac in terms of benefit and concomitant risk with other non-steroidal anti-inflammatory drugs (NSAIDs) as well as with coxibs [1]. Diclofenac at 150 mg/day has better pain relief than celecoxib, naproxen, and ibuprofen, but diclofenac at 100 mg/day has benefits similar to those of the comparators. Furthermore, diclofenac is similar to the coxibs (and maybe worse than etoricoxib) in terms of gastrointestinal (GI) risk and better than that observed with naproxen or ibuprofen treatment; interestingly, in this data set including 146,524 patients from 176 randomized controlled trials (RCTs), there was no difference between therapies regarding cardiovascular (CV) risk.

We are frequently bombarded by new reports which often conflict. These are either observational data sets or yet another meta-analysis of multiple RCTs of varying lengths, with details regarding the risk of using an NSAID. Unfortunately, almost all of these studies present evidence regarding the drug's risk of either a CV event or a GI event and do not compare the balance of risk between these CV or GI events for any one drug in a single report, nor have the same studies assessed efficacy at the same time. The Coxib and Traditional NSAID Trialists’ Collaboration developed a meta-analysis of 280 RCTs of NSAIDs versus placebo (124,513 patients) and 474 trials of one NSAID versus another (229,296 patients), focusing on risk for major CV events (non-fatal myocardial infarction, non-fatal stroke, or CV death), all-cause mortality, heart failure, and upper GI complications (perforation, obstruction, or bleed) [2]. That report is informative compared with earlier data sets since we learn that naproxen might be safer for patients with CV risk but that it is one of the worst NSAIDs in terms of risk for a major GI complication. By providing similar evidence but including data regarding benefit would give far better information for the clinician to choose a drug for any one patient while considering that patient’s unique risk factors.

More evidence was contributed by a US Food and Drug Administration Arthritis Advisory Committee meeting convened to determine whether naproxen was safe in terms of CV risk [3]. There was no agreement that naproxen has been proven to be safe at this time. The only added information was the recognition that the risk for a CV event may be earlier in treatment than previously thought. Thus, this earlier CV risk mirrors the early risk for GI ulcer damage reported to be present within seven days of systemic therapy, even in normal human volunteers endoscoped for that purpose [4].

For clinicians, it must be difficult to consider this conflicting evidence as it has evolved. Achieving adequate pain relief is an important treatment goal. There is evidence that chronic pain, particularly severe pain such as pain resulting in inactivity, is associated with increased all-cause mortality [5–8]. Some well-designed observational studies fail to corroborate increased CV risk with NSAIDs and suggest that long-term treatment with NSAIDs or coxibs is associated with a substantially reduced incidence of CV events and all-cause mortality perhaps linked to increased activity with adequate pain relief [7, 8].

In some studies, NSAIDs and coxibs have lower rates of significant harm than opioids in large matched cohorts [9]. Despite this evidence, some developers of treatment guidelines have chosen to suggest opioids as alternative therapies for patients, implying that opioids would be safer than the NSAIDs [10]. By suggesting opioid therapy as an alternative, these guideline developers have chosen to ignore the ample literature demonstrating serious risks for many patients using opioids. These risks include dysphoria, which can lead to increased patient falls and consequent hip fracture in older patients. A large propensity-matched study reported the incidence of fracture with opioids to be five times higher than that with NSAIDs in older adults, and hospital admission for adverse events and all-cause mortality were also higher with opioids [5, 9]. A meta-analysis of RCTs of NSAID use indicates a 45 % increased risk of a CV event compared with placebo and this translates to a 0.3 % increased absolute risk per year compared with a background risk of about 1 % per year in the at-risk population [5].

A clinician needs clear guidance to be able to choose the right type of therapy for the individual patient. Pain needs to be treated with therapies which provide substantive benefit. For patients with chronic pain, there is a problem of confounding by indication. There is an increased risk for CV events in untreated patients due to decreased physical functioning and consequent increased inactivity. [5]. Balancing the risk of no treatment with the competing potential risks of various therapies is the clinician’s responsibility, and the report by van Walsem and colleagues [1] helps to improve our understanding of exactly what these competing risks may mean for our patients.

## Abbreviations

CV: Cardiovascular
GI: Gastrointestinal
NSAID: Non-steroidal anti-inflammatory drug
RCT: Randomized controlled trial
